# Supplementary material for: Powerful and Real-Time Quantification of Antifungal Efficacy against Triazole-Resistant and -Susceptible Aspergillus fumigatus Infections in Galleria mellonella by Longitudinal Bioluminescence Imaging
Source: Microbiol Spectr. 2023 Jul 19;11(4):e00825-23. doi: 10.1128/spectrum.00825-23 (PMC10433797; doi:10.1128/spectrum.00825-23)
Supplement: Supplemental file 1 — Fig. S1 to S4. Download spectrum.00825-23-s0001.docx, DOCX file, 0.6 MB [file spectrum.00825-23-s0001.docx]

Supplementary figures


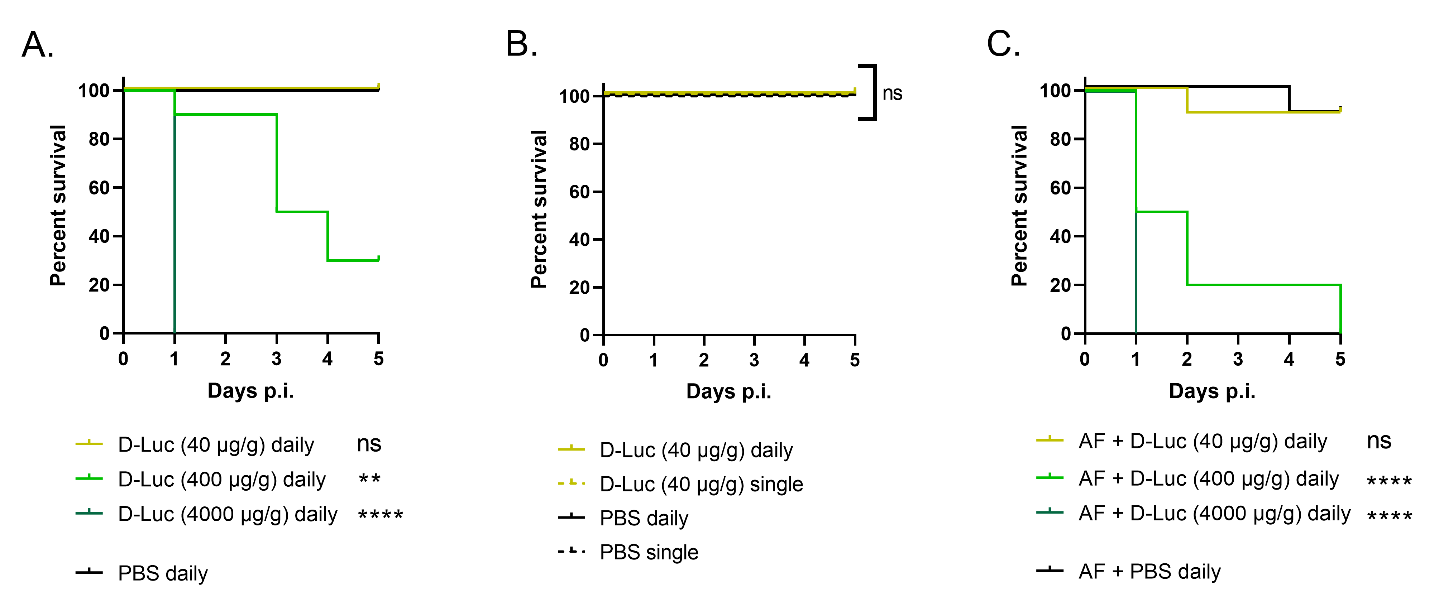


**Figure S1: Tolerability of substrate dose and frequency for *in vivo* BLI in *G. mellonella*: Survival studies.** Tolerability of **(A)** different doses and **(B)** frequencies of d-luciferin injections compared to PBS in healthy larvae as measured by survival. **C)** Tolerability of different doses of d-luciferin injections compared to PBS in larvae infected with 10^5^ conidia of AF TR_34_/L98H as measured by survival. Data are mean percent survival per group (*n* =10). ** *P*<0.01, *****P*<0.0001; ns = non-significant.


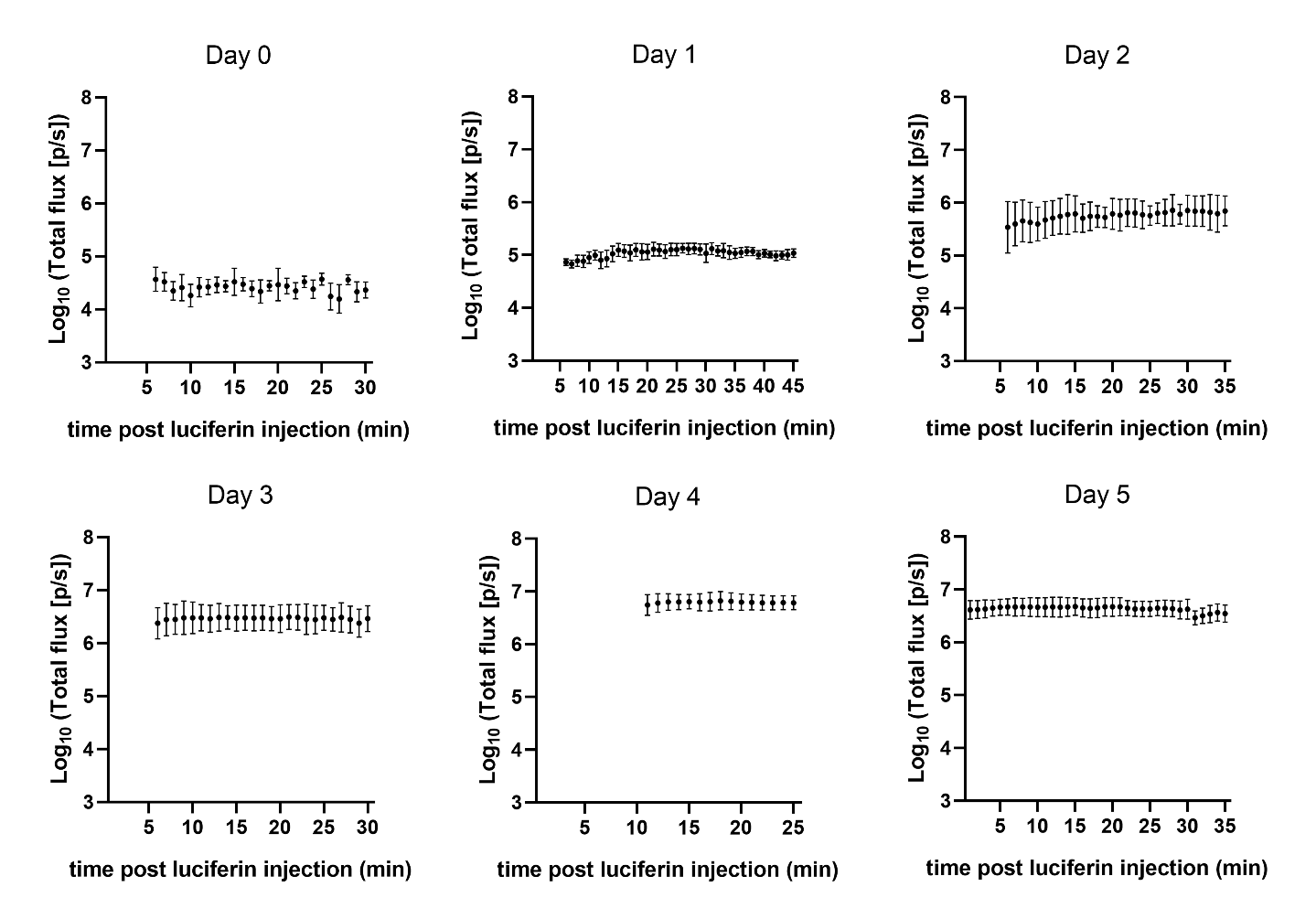


**Figure S2**: **Kinetics of *in vivo* photon flux in *G. mellonella* after substrate injection.** BLI signal in infected larvae (10^5^ conidia AF TR_34_/L98H) over time after d-luciferin injection from day 0 (baseline) until day 5 post infection. Data are mean ± SD (*n* = 10).


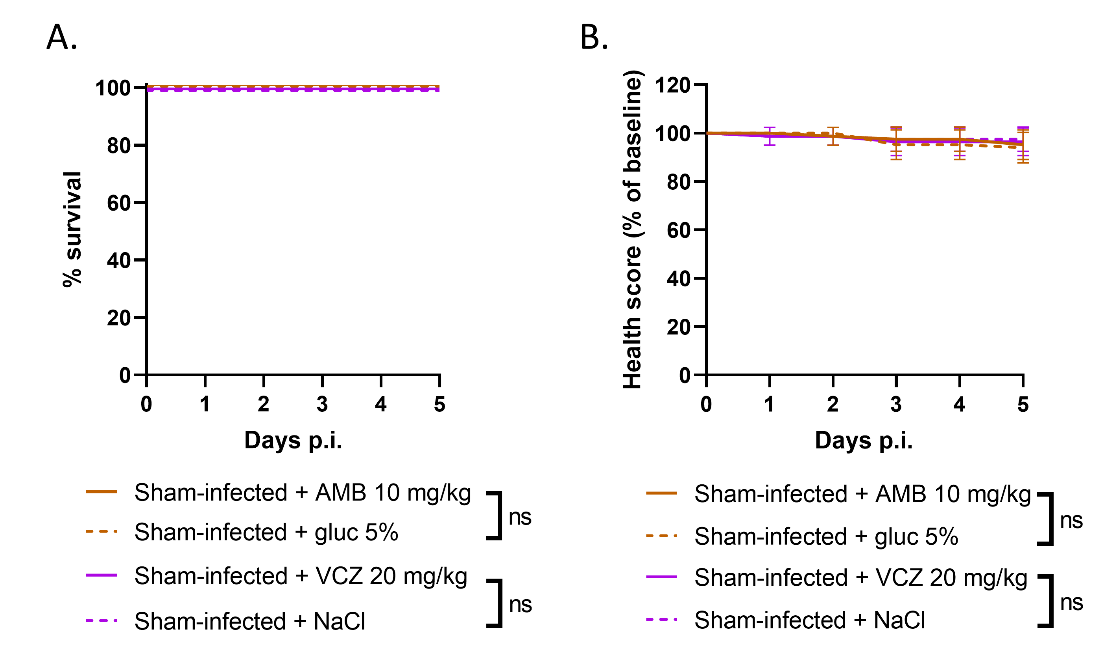


**Figure S3: Daily AMB and VCZ treatment are well tolerated by *G. mellonella* larvae. A)** Survival and **B)** health score of mock-infected larvae receiving daily treatment (vehicle) over 5 days pi. Data are mean (± SD) (*n* = 10). ns = non-significant.


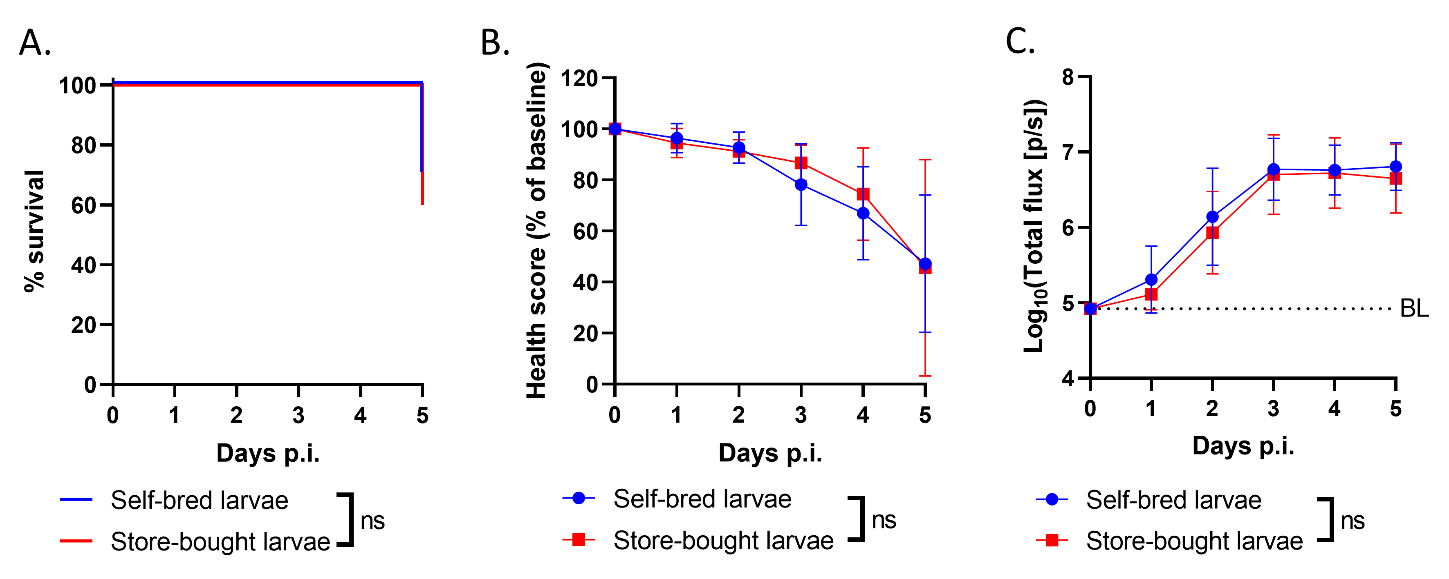


**Figure S4: Similar survival, health score and *in vivo* BLI in self-bred and store-bought *G. mellonella* larvae. A)** Survival, **B)** health score and **C)** *in vivo* BLI in larvae from our in-house breeding compared to larvae purchased from a non-research grade commercial breeder. Healthy larvae were infected with 10^5^ conidia AF WT and followed until 5 days pi. Data are mean (± SD) (*n* = 10). ns = non-significant. BL (baseline) represents background BLI signal (dotted line).
